# Bibliometric Analysis and Visualization of Academic Procrastination

**DOI:** 10.3389/fpsyg.2021.722332

**Published:** 2021-10-18

**Authors:** Xue Tao, Hafiz Hanif, Hamsa Hameed Ahmed, Nader Ale Ebrahim

**Affiliations:** ^1^College of Foreign Languages, Baoji University of Arts and Sciences, Baoji, China; ^2^Fakulti Pembangunan Manusia, Universiti Pendidikan Sultan Idris, Tanjong Malim, Malaysia; ^3^Fakulti Pembangunan Manusia–Jabatan Pengajian Pendidikan, Universiti Pendidikan Sultan Idris, Tanjong Malim, Malaysia; ^4^Research and Technology Department, Alzahra University, Tehran, Iran; ^5^University of Malaya, Kuala Lumpur, Malaysia

**Keywords:** bibliometric analysis, academic procrastination, bibliometrix tool, VOSviewer, the trend

## Abstract

Numerous students suffer from academic procrastination; it is a common problem and phenomenon in academic settings. Many previous researchers have analyzed its relationships with other factors, such as self-regulation and academic success. This paper aims to provide a full outline of academic procrastination and explore the current hot spots and trends. Bibliometrix and VOSviewer were used to conduct quantitative analysis. The data was collected from the Web of Science core collection database, which contains 1,240 articles from the years 1938 to 2021. The analysis shows that the publication of articles on academic procrastination has been rapidly increasing since 1993. In terms of the most influential countries and institutions, the United states took a prominent lead among all countries, and the most productive institutions in this area were the University of Washington and University of California, Los Angeles. By analyzing the authors, we see that most authors like working with a few collaborators, leading to main groups of authors, such as Murat Balkis and June J. Pilcher. The most frequently cited author was Esther D. Rothblum. Based on the co-citation journals network, *Personality and Individual Differences* was the prolific and influential journal referring to the number of citations and articles it received. The VOSviewer tool identified the hot spots of academic procrastination, which were mainly distributed as follows: (a) procrastination, (b) academic procrastination, (c) self-regulation, (d) academic performance, and (e) motivation. Therefore, this paper is helpful for scholars and practitioners to know the trend of academic procrastination research comprehensively.

## Introduction

Academic procrastination is considered problematic to academic success and a self-regulated failure among university students (Zhang et al., 2018; Hailikari et al., 2021). It is necessary to study the trend of academic procrastination comprehensively to manage to reduce it.

### The Emergence of Academic Procrastination

Procrastination is a shared learning phenomenon, which usually leads to a delay in learning tasks and activities, thus decreasing the quality and quantity of students' learning. A prevalent and universal problematic behavior (Gao et al., 2021), “academic procrastination” appears when it relates to academic settings (Aznar-Diaz et al., 2020). There are various definitions of academic procrastination as it occurs in different fields. It means putting off tasks or failing to finish them (Aznar-Diaz et al., 2020) or delaying academic studies purposefully (Schraw et al., 2007; Chen, 2019). It can also be described as a failure of self-regulation, incapable of supervising, regulating, and playing with the preferred criteria for controlling impulses, emotions, task performance, and thoughts (Wolters, 2003; Balkis and Duru, 2016; Zhang et al., 2018).

Academic procrastination is a deliberate postponement of academic assignments, which is a common phenomenon among students (Mohammadi Bytamar et al., 2020) despite awareness of its consequences. It is reported that about 80–95% of college students were engaged in such problematic behavior (Rahimi et al., 2016), and it is estimated that 90% of college students procrastinate for more than an hour every day (Klassen et al., 2008; Rahimi et al., 2016).

### The Impediment of Academic Procrastination

Academic procrastination, which is common among students at all levels of education, is associated with negative consequences, such as failure (Sarid et al., 2021). Academic procrastination does not exist as an independent phenomenon (Solomon and Rothblum, 1984; Steel, 2007; Rakes and Dunn, 2010; Chow, 2011; Janssen, 2015; Ziegler and Opdenakker, 2018). In fact, many researchers acknowledge its negative association with self-regulated learning strategies and academic success.

Lots of previous research reveals that academic procrastination is a severe threat to the academic success of students (Kim and Seo, 2015; Steel and Klingsieck, 2016; Jin et al., 2019). It hinders academic success by its negative effects on the learning quantity and quality and results in lower achievement in academic settings (Howell and Watson, 2007; Batool, 2020).

As an adverse effect of the failure of self-regulatory learning, academic procrastination (Howell and Watson, 2007; Cerezo et al., 2016) leads to learning activities being delayed for students in academic settings. Prior studies also claim that failure to regulate oneself is one of the main reasons causing procrastination (Steel, 2007; Steel and Klingsieck, 2016; Jin et al., 2019).

### The Present Study

Scholars have conducted much research on academic procrastination. However, most of the articles start from a single point of view, and some scholars focus on its relationship with other strategies or factors (Aznar-Diaz et al., 2020; Limone et al., 2020; Gao et al., 2021), such as its relationship with self-regulated strategies (Ziegler and Opdenakker, 2018), academic achievement (Batool, 2020), and life satisfaction (Çikrikçi and Erzen, 2020). Some scholars focus on interventions to reduce academic procrastination (Hanger et al., 2019; Krispenz et al., 2019), and a few studies focus on reviewing the literature on the subject matter (Pinxten et al., 2019; Wernecke et al., 2019; Svartdal et al., 2020). The present literature is found to be lacking in a comprehensive review of academic procrastination and the highlights of the topic in recent years. Academic procrastination is connected to other areas, including education, psychology, psychiatry, management, computer science, economics, and social science. A bibliometric analysis is necessary to understand the progress and hot spots of academic procrastination comprehensively.

Bibliometrics is a unique R-tool for comprehensive scientific map analysis, developed using statistical computation and graphic R languages following a logical bibliometric workflow, including all significant bibliometric analysis methods. It is used to evaluate a specific subject's quantity and development trend (Chen et al., 2018, 2021; Hao et al., 2018). It is also a valuable assessment of books, articles, and other publications in a particular field with the passage of time (van Raan, 2005). Bibliometrics can help researchers to observe the performance of various subjects. This paper uses R-tool and VOSviewer to generate maps and carry out a systematic and comprehensive analysis of academic procrastination.

This study is structured as follows. First, data sources and the methods used are reviewed. Second, bibliometric analysis leverages the results from the following six aspects: publication trend, classification analysis, author co-citation network and author collaboration network, cooperation analysis of institutions and countries, analysis of journal co-citation, and analysis of cited references. Third, hot spots of the research and new trends in particular areas are then summarized. Fourth, conclusions as well as future directions of research are put forth. Compared with the traditional literature review method, this paper comprehensively highlights new trends in academic procrastination. The systematic collection, collation, and analysis of research papers related to academic procrastination serve as a guideline for future researchers who are interested in exploring academic procrastination. This study seeks to answer the following research questions: (1) What are the subject categories and general publication trends? (2) Which organizations/institutions, authors, or countries are involved actively in academic procrastination study? (3) Which journals are cited most in the area of academic procrastination research study? (4) What are the highly cited references in academic procrastination study? (5) What are the emerging trends and the hot research topics in academic procrastination articles?

## Methodology

### Data Collection

**Figure 1:**
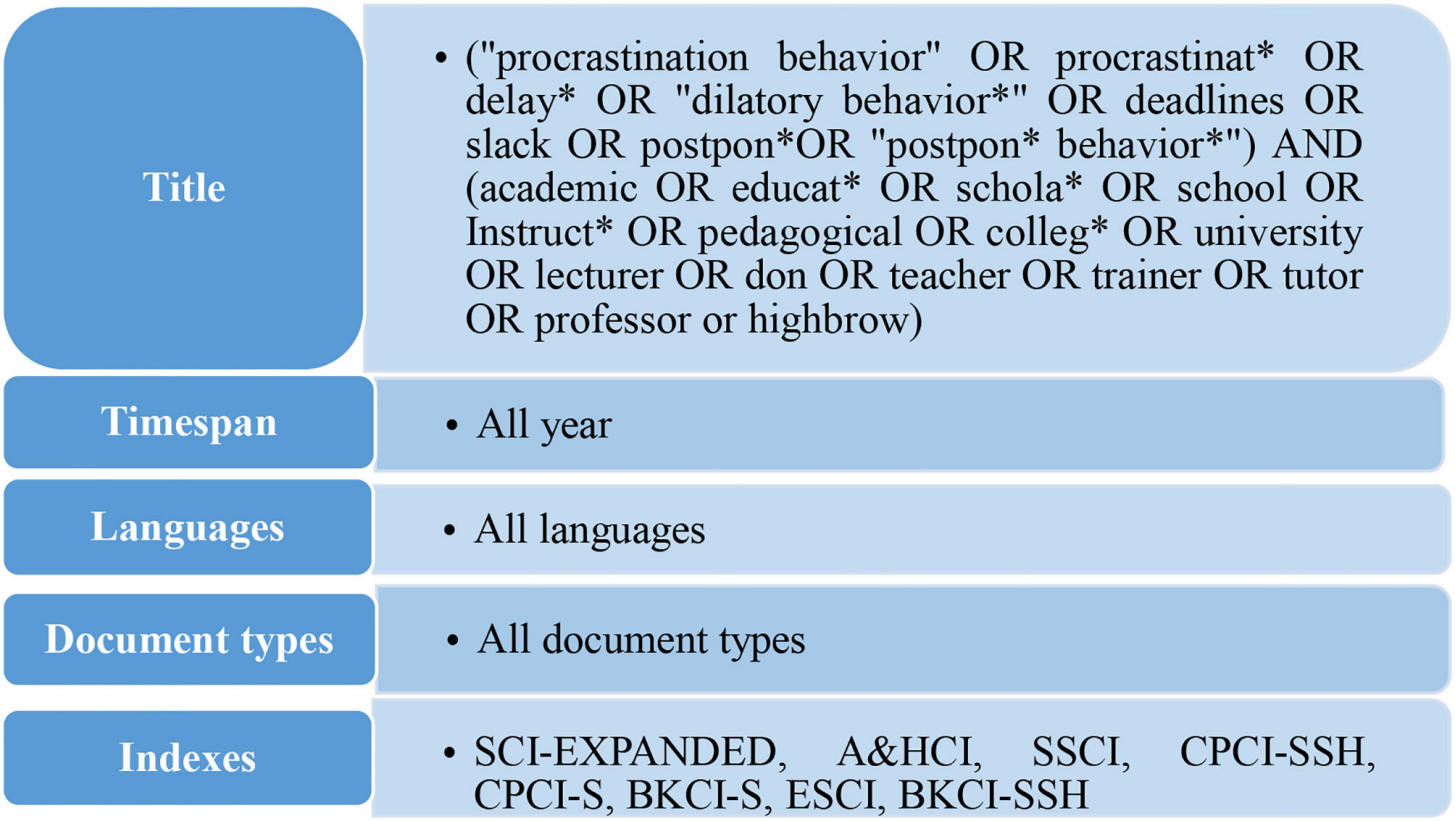
Data queries.

There are two well-known bibliometric databases: Clarivate Analytics' Web of Science (WoS) and SCOPUS (Aghaei Chadegani et al., 2013). Besides these two reliable sources, new bibliometric databases have recently become available, such as Google Scholar, Microsoft Academic, Dimensions, and the Open Citations Index of CrossRef open DOI-to-DOI citations (COCI) (Martin-Martin et al., 2020). To select the proper database for the current study, a title search with the same syntax was carried out on both databases. There were 1,240 documents on WoS and 1,202 documents on SCOPUS. The results indicate that the WoS database was more successful in covering the title search compared with SCOPUS. Therefore, for the current study, data has been retrieved from the WoS database due to its high quality standards and wider coverage, which can easily refine and filter queries (Elaish et al., 2019). There are 1,240 articles analyzed in this study. The method of querying is showed as follows (see Figure 1).

### Data Analysis

In the present study, the Bibliometrix tool with the graphic R language (version R x64 4.0.3) was used to analyze the selected publications on the field of academic procrastination comprehensively and clearly, such as the hot research topics, the paper quality, sources, best authors, most cited keywords, and research categories from the year 1938 to January of 2021.

All information on the data has been exported into Microsoft Office Excel to analyze. In addition, VOSviewer is a visualizing and constructing software tool for bibliometric papers (van Eck and Waltman, 2010). The co-occurrence network in terms of keywords unit; the bibliographic coupling network in terms of authors, countries, sources, and organizations unit; and the co-citation network in terms of cited authors and cited references unit were imported into VOSviewer for further detailed analyses.

## Results

### Analysis of Publication Outputs

**Figure 2:**
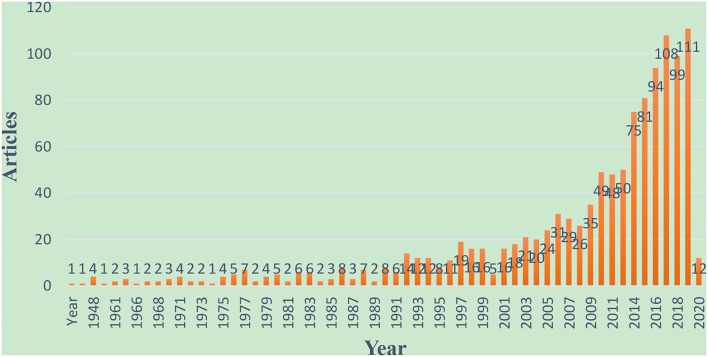
The annual publication rate of academic procrastination research.

The publication trends in each year can help in understanding academic procrastination's stages of development (Guo et al., 2021). As presented in Figure 2, overall, the main trend of annual publications is on the rise. The total number of articles published in 1938–1992 was 109, and among these years, the articles in each year were no more than 10; the years 1939–1947, 1949–1956, 1958–1960, 1963–1965, and 1969 have no articles at all. After 1993, the number of articles shows a continuous rising trend: in the year 2007, there were 31 articles published; in the year 2013, there were 53 articles published; in the year 2020, there were 111 articles published. There were roughly two periods in publication development. The first one began from 1938 to 1992, and the number of publications about academic procrastination were scarce, which reflects that academic procrastination was not very popular at that time. In the second one from 1993 to 2021, the number of academic procrastination articles published increased rapidly, indicating the increasing attention and research on academic procrastination. To some extent, it can be seen that researchers or scholars begin to attach importance to the problem of academic procrastination.

### Analysis of Categories

According to WoS classification, a literature review on academic procrastination is able to reflect the research development level of a particular discipline in a particular period and display research objects that are receiving more and more attention as time goes by Hamidi and Ramavandi (2020). Academic procrastination includes about 25 field classifications in the WoS database. Table 1 shows the top five subject categories, which includes educational research (201 articles, 16.21%), psychology multidiscipline (118 articles, 9.52%), psychology educational (90 articles, 7.26%), clinical neurology (86 articles, 6.94%), and rehabilitation (59 articles, 4.76%). The subject categories distribution shows that key points in these subjects gained high priority in the studies. The publication number in different categories shows the trends of academic procrastination study in various fields.

**Table 1:** Top 5 subject categories based on publications.

**Subject category**	**1938–2021**	**%/of 1,240**
Education educational research	201	16.21
Psychology multidiscipline	118	9.52
Psychology educational	90	7.26
Clinical neurology	86	6.94
Rehabilitation	59	4.76

### Analysis of Institutions and Countries

**Figure 3:**
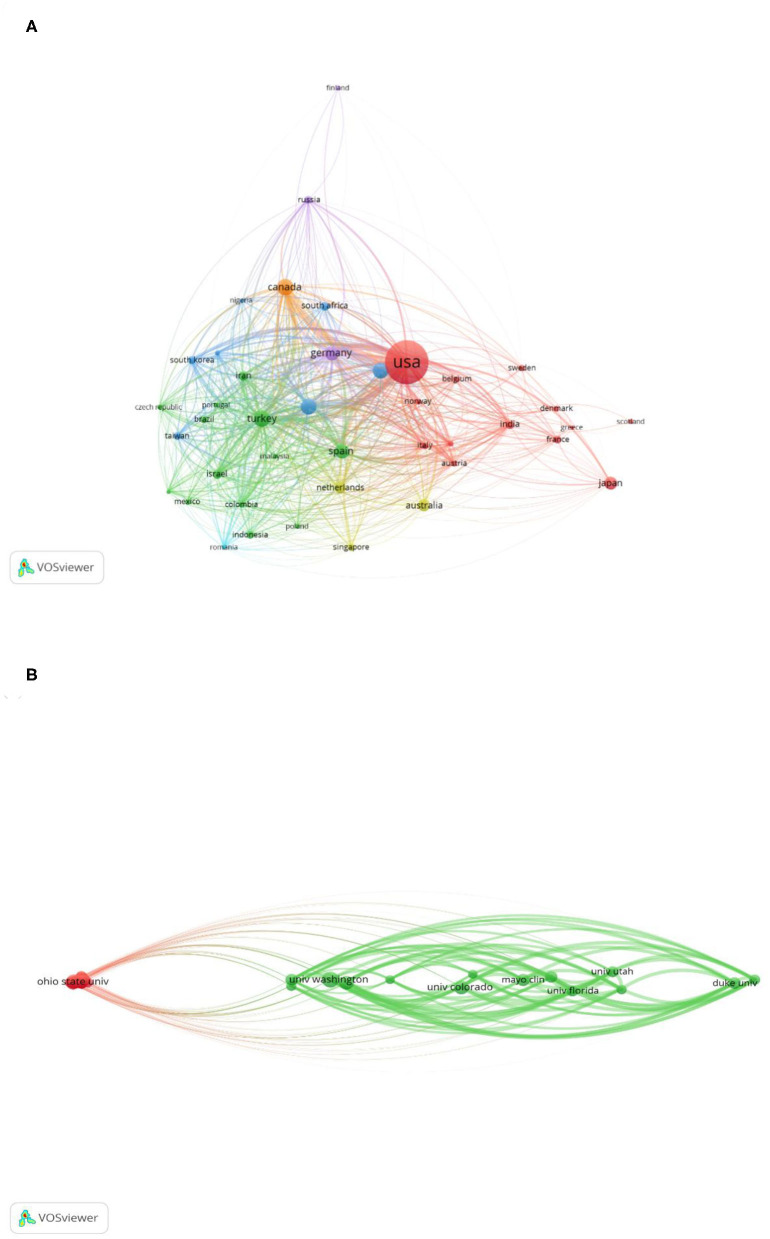
The visualized map of organizations/countries participating in academic procrastination study: **(A)** Mapping of major countries participating in academic procrastination study. **(B)** Mapping of organizations involved in academic procrastination study.

The study of national distribution is helpful to know the geographical and spatial distribution of papers. The cooperation between countries in academic procrastination study is shown in Figure 3A; the VOSviewer was used to plot the visualization of main countries. The size of the nodes represents the difference in the number of articles published in different countries. The larger node represents more published articles. International institutions; and countries' cooperation and exchanges among institutions and countries must be strengthened.

As can be seen from Table 2, the United States has the largest number of published articles, about 460, which accounts for 37.10%, followed by Canada's 64 articles, which account for 5.16%, and Turkey's 62 articles. There are 899 articles published by the top 10 countries, accounting for 72.50% of the total 1,240 articles on academic procrastination research, and other countries on academic procrastination research account for only 27.50%.

**Table 2:** Top 10 countries based on publications.

**Rank**	**Country**	**Publication**	**%/of papers**	**TC**	**TC/P**
1	United States	460	37.10	9,749	24.619
2	Canada	64	5.16	1,672	35.574
3	Turkey	62	5.00	436	8.226
4	England	57	4.60	915	22.317
5	China	56	4.52	310	5.082
6	Spain	49	3.95	147	4.455
7	Germany	48	3.87	511	12.775
8	Australia	37	2.98	339	16.143
9	Japan	37	2.98	275	10.185
10	Netherlands	29	2.34	317	16.684

*TC, the total number of citations a country has received; TC/P, the average number of citations per paper in a country*.

A country's total number of citations is another significant indicator of a country's scientific impact in the field of academic procrastination. Notably, the total number of citations for the United States is significantly higher than for any other country (9,749 citations in all). The United States analyzed the causes, development, negative effects, and elimination of academic procrastination actively. In terms of the TC/P and TC values, the United States, Canada, England, Turkey, and Germany significantly contribute to the full effort. Canada's TC/P was as high as 35.574 although Spain has relatively low TC and TC/P.

As shown in Figure 3B, the VOSviewer software was used to draw a visualization map of the major organizations. Table 3 lists the top 10 most productive institutions in publishing. The institutions that bring influential effects are the University of Washington (25 articles); University of California, Los Angeles (17 articles); McGill University (14 articles); The Ohio State University (14 articles); University of Colorado (14 articles); and Mayo Clinic (14 articles). In these institutions, eight came from the United States, and the remaining two are from Turkey and Canada, which show that the United States made a remarkable contribution to the area of academic procrastination.

**Table 3:** Top 10 institutions based on publications.

**Rank**	**Institution**	**Publication**	**Country**
1	University of Washington	25	United States
2	University of California Los Angeles	17	United States
3	McGill University	14	Canada
4	Ohio State University	14	United States
5	University of Colorado	14	United States
6	Mayo Clinic	13	United States
7	Yale University	13	United States
8	Pamukkale University	12	Turkey
9	University of California San Francisco	12	United States
10	University of Wisconsin	12	United States

### Analysis of Author Co-citation Network and Author Collaboration Network

**Figure 4:**
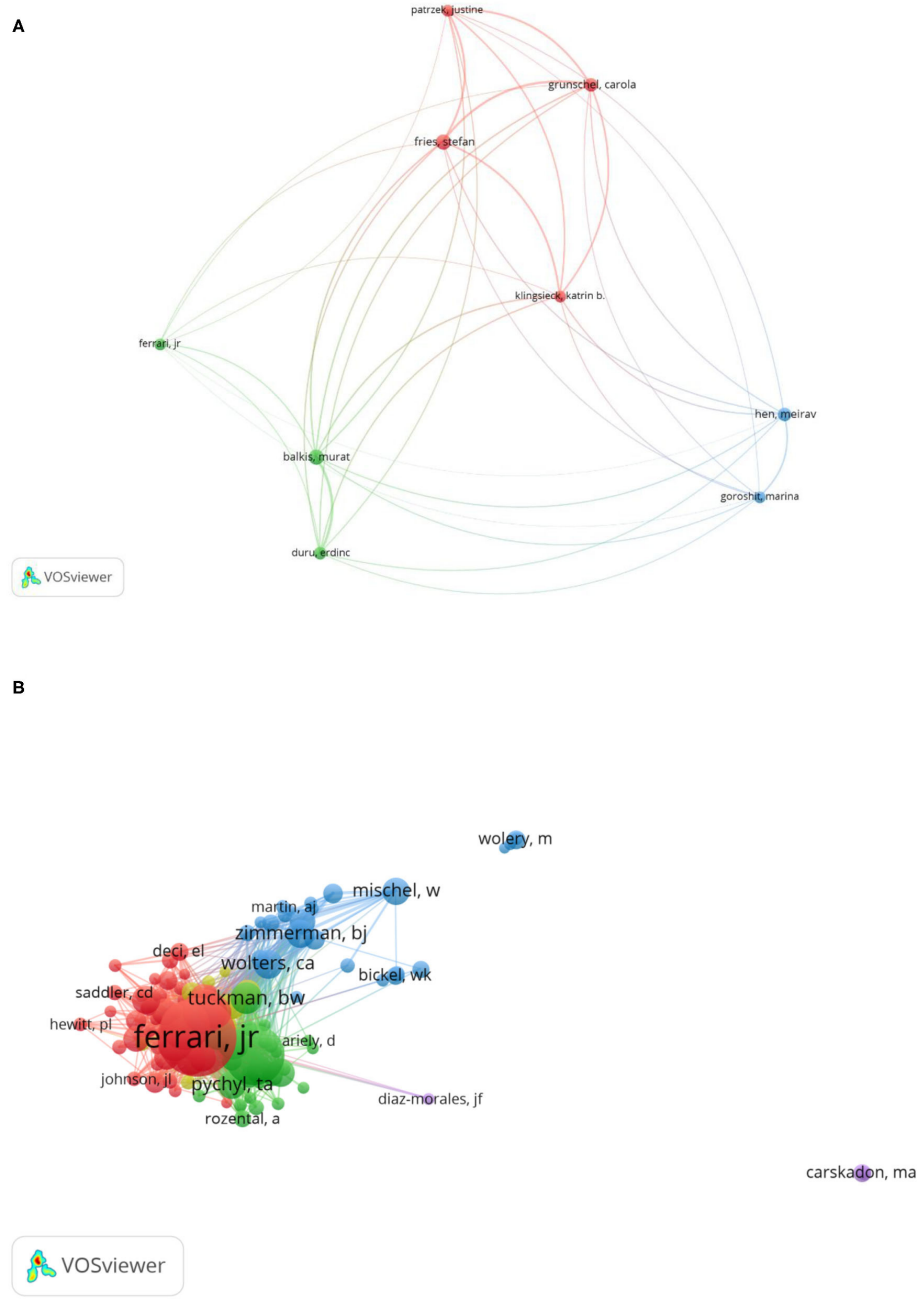
The visualization map of main authors participating in academic procrastination research: **(A)** Mapping of main authors participating in academic procrastination research. **(B)** Mapping of co-cited authors participating in academic procrastination research.

The author of the research plays a critical part in reflecting research competence and evaluating progress in the academic area. In Figure 4A, cooperation among authors is mapped vividly in the field of academic procrastination, using the VOSviewer software to create the visualization map of the main authors. It is worth noting that most authors like working with a small number of collaborators, which led to few links between the major authors. From 1938 to 2021, the top 10 most prolific authors and their affiliates are listed in Table 4. In terms of their published articles, the authors who come from Germany (17 articles) dominate the published articles. Other authors with prominent publications include Murat Balkis (nine articles) and Stefan Fries (nine articles). The unremitting efforts of these authors contribute to the promotion of research on academic procrastination. For instance, the author Pilcher from Germany studies delaying school, which is caused by sleep response (Pilcher, 2016). Balkis and Duru investigates the role of self-regulated failure on procrastination as well as the impact of procrastination on affective well-being and academic life satisfaction (Balkis and Duru, 2016). Grunschel et al. reveals that, through the mediation of academic procrastination, the relationships among motivational regulation strategies, students' academic performance, and affective/cognitive well-being are positive and indirect (Grunschel et al., 2016). A study from Herndon et al. shows that students with a high tendency for gratification delaying and a low tendency for violent action achieved great scores on the tests (Herndon et al., 2015). Ozer et al. uses cognitive interventions on the procrastination of students to evaluate a short-term treatment plan (Ozer et al., 2013).

**Table 4:** Top 10 most productive authors in academic procrastination research: 1938–2021.

**Rank**	**Publications**	**Author**	**Institution**	**Country**
1	17	June J. Pilcher	College of Behavioral, Social and Health Sciences	Germany
2	9	Murat Balkis	Pamukkale University	Turkey
3	9	Stefan Fries	Bielefeld University	Germany
4	7	Héfer Bembenutty	Queens College of the City University of New York	United States
5	7	Joseph R. Ferrari	DePaul University	United States
6	7	Carola Grunschel	Bielefeld University	Germany
7	7	Meirav Hen	Telhai Academic College	Israel
8	6	Erdinç Duru	Pamukkale University	Turkey
9	6	Fred M. Kusumoto	Mayo Clinic	United States
10	6	Annette Majnemer	University of Montreal	Canada

As for analysis of co-authorship, in Figure 4B, the VOSviewer software plotted the mapping of the co-cited authors, and they are represented by their tags, by default, as well as by a circle. The higher the weight of the co-cited authors, the larger the label and the circle of the item. The cluster of the items determined the color of the item. Links were represented by the lines between items. Table 5 lists the top 10 most frequently cited authors. The most frequently cited authors were Esther D. Rothblum (frequency 201) and L. J. Solomon (frequency 201), who examine how often procrastination of college students and the causes of procrastination action and academic assignments (Solomon and Rothblum, 1984). The second is Klassen et al. (frequency 110), who conducted two studies to investigate academic procrastination and motivation (Klassen et al., 2010). The third was Milgram et al. (frequency 82), who found that avoidant procrastination is common to delay actions in both non-academic life routines and academic assignments (Milgram et al., 1998). Fourth place went to Senecal et al. (frequency 79), who investigated the function of self-regulation in predicting academic procrastination (Senécal et al., 1995).

**Table 5:** Top 10 cited authors and their highly cited articles: 1938–2021.

**Rank**	**Frequency**	**Year**	**Author**	**Highly cited references**
1	201	1984	Esther D. Rothblum	Academic procrastination-frequency and cognitive-behavioral correlates
2	201	1984	L. J. Solomon	Academic procrastination-frequency and cognitive-behavioral correlates
3	110	2010	Robert M. Klassen	Academic procrastination in two settings: motivation correlates, behavioral patterns, and negative impact of procrastination in Canada and Singapore
4	82	1998	N. N. Milgram	Procrastination, generalized or specific, in college students and their parents
5	79	1995	C. Senecal	Self-regulation and academic procrastination
6	78	2008	Lindsey L. Krawchuk	Academic procrastination of undergraduates: low self-efficacy to self-regulate predicts higher levels of procrastination
7	74	2007	Stefan Fries	Individual values, learning routines, and academic procrastination
8	73	2013	Joseph R. Ferrari	Sex, education, and procrastination: an epidemiological study of procrastinators' characteristics from a global sample
9	72	2015	Eun Hee Seo	The relationship between procrastination and academic performance: a meta-analysis
10	71	2013	Katrin B. Klingsieck	Procrastination when good things don't come to those who wait

### Analysis of Co-citation Journals

To identify the core journals in an area, one must focus on the frequency and the number of article citations. Table 6 shows that *Personality and Individual Differences* published the most, followed by *Learning and Individual Differences*. Notably, *Personality and Individual Differences* is also cited the most (993 citations). The results suggest that *Personality and Individual Differences* plays an important role in academic procrastination research, which is far more valuable than any other journal. The H-index can be used to assess the impact of journals. According to the H-index, *Personality and Individual Differences* went the first rank with the highest H-index of 16, which has the greatest effect on academic procrastination study. A classification of the top five journals in terms of subjects discovered that *Psychology and Social* received attention more than other journals. Higher citations and more publications in these journals indicatie that these journals occupy the most popular places in the area.

**Table 6:** Top 5 productive journals in academic procrastination: 1938–2021.

**Rank**	**Journal**	***P_a_***	**%/of papers**	**TC_**b**_**	**TC/P_**c**_**	**H-index**	**IF**	**Subject**
1	Personality and individual differences	22	1.77%	993	45.14	16	2.310	Psychology, social
2	Learning and individual differences	19	1.53%	453	23.84	12	1.916	Psychology, educational
3	Frontiers in psychology	17	1.37%	68	4.00	5	2.067	Psychology, multidisciplinary
4	International journal of psychology	14	1.13%	1	0.07	1	1.255	Psychology, multidisciplinary
5	Psychological reports	14	1.13%	351	25.07	9	1.535	Psychology, multidisciplinary

*P_a_, the total publications of a journal in academic procrastination; TC_b_, the total number of citations for a journal; TC/P_c_, the average number of citations per paper for a journal; IF, a 5-year impact factor, based on data from the 2019 edition of Journal Citation Reports^R^ in WoS; H-index: according to analysis of the Bibliometix tool*.

**Figure 5:**
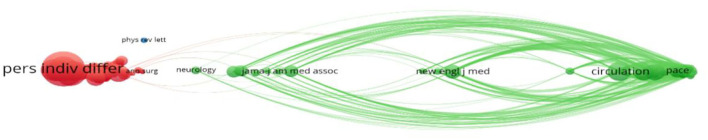
The time zone view of the co-citation journal network: 1938–2021.

By analyzing the co-citation journals, it is found that the distribution of highly cited journals is reflected vividly in the academic procrastination area. In Figure 5, the VOSviewer software was used to draw the time zone view of the network of co-cited journals.

### Analysis of Cited References

Table 7 lists the top 10 reference articles based on the number of citations. The cited references analysis helps researchers to understand the internal relationships among organizations, authors, and countries. In the field of academic procrastination, the most cited reference is from author Laura J. Solomon, “Academic Procrastination: Frequency and Cognitive-Behavioral Correlates.” The second comes from Piers Steel, “The Nature of Procrastination: A Meta-Analytic and Theoretical Review of Quintessential Self-Regulatory Failure” (Steel, 2007). The third is Dianne M Tice, “The Nature of Procrastination: A Meta-Analytic and Theoretical Review of Quintessential Self-Regulatory Failure” (Tice and Baumeister, 1997). The fourth is Lay, “At Last, My Research Article on Procrastination” (Lay, 1986). Their research is considered the base of the area due to the high co-citation and great impact. In terms of geographical distribution, six authors—Laura J. Solomon, Dianne M. Tice, Esther D. Rothblum, A. Ellis, Gregory Schraw, and Joseph R. Ferrari—came from the United States, which shows that the United States led the research of academic procrastination.

**Table 7:** Top 10 cited references.

**Citation**	**Year**	**Author(s)**	**Title**	**Source**	**Country**
201	1984	Laura J. Solomon	Academic procrastination: frequency and cognitive-behavioral correlates	Journal of counseling psychology	United States
173	2007	Piers Steel	The nature of procrastination: a meta-analytic and theoretical review of quintessential self-regulatory failure	Psychological bulletin	Canada
96	1997	Dianne M Tice	Longitudinal study of procrastination, performance, stress, and health: the costs and benefits of dawdling	Psychological science	United States
90	1986	Clarry H. Lay	At last, my research article on procrastination	Journal of research in personality	Canada
83	1986	Esther D. Rothblum	Affective, cognitive, and behavioral differences between high and low procrastinators	Journal of counseling psychology	United States
76	1977	Ellis A.	Overcoming procrastination	Book	United States
71	2007	Gregory Schraw	Doing the things we do: a grounded theory of academic procrastination	Journal of educational psychology	United States
68	1995	Joseph R. Ferrari	Procrastination and task avoidance: theory, research, and treatment	Book	United States
66	2003	Wendelien van Eerde	A meta-analytically derived nomological network of procrastination	Personality and individual differences	The Netherlands
61	2008	Robert M. Klassen	Academic procrastination of undergraduates: low self-efficacy to self-regulate predicts higher levels of procrastination	Contemporary educational psychology	Canada

## Hot Research Topics and Emerging Trends

### Hot Research Topics

**Figure 6:**
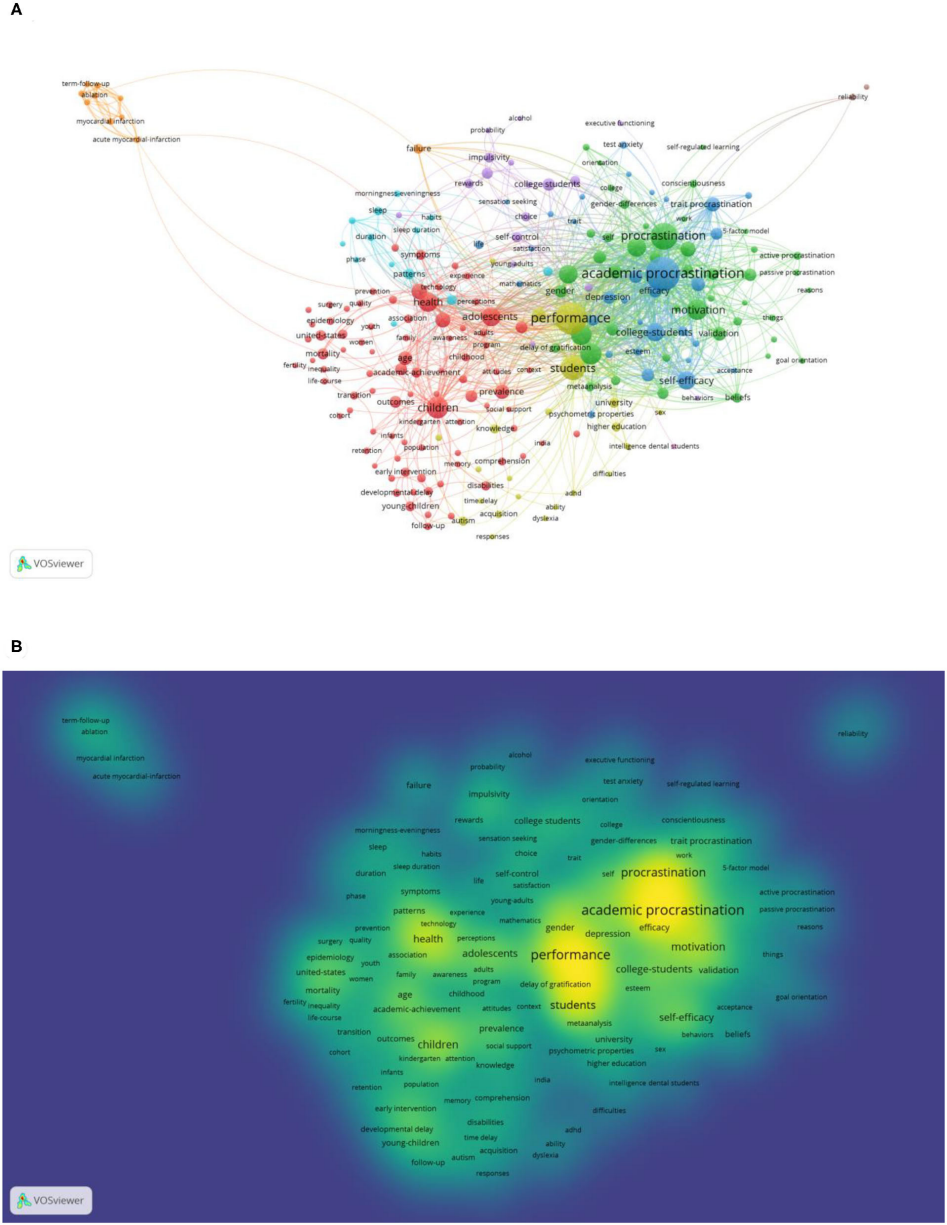
Knowledge domain map of keyword co-occurrence network related to academic procrastination research: **(A)** network visualization map based on article weights; **(B)** density visualization map based on article weights.

Analyzing keyword frequency in the research field and specific discipline could give a rough overview of the field of the academic procrastination. As shown in Figure 6A, the VOSviewer software was used to draw the keyword co-occurrence network.

Cluster 1 (Red): The largest node was children, which relates to 85 keywords, including adolescents, health, age, young children, youth, and education. This cluster focuses more on the different education stages of academic procrastination.

Cluster 2 (Green): The largest node was procrastination, which is mostly associated with keywords, such as academic procrastination, academic performance, trait motivation, college, academic achievement, and self-efficacy. This branch mainly focuses on different types of procrastination and the important factors related to them.

Cluster 3 (Blue): The largest node is academic procrastination. This cluster is interested in the phenomenon of procrastination and the related elements appearing in academic settings, such as college students, self-efficacy, stress, trait procrastination, self-esteem, and optimism. Previous research shows that academic procrastination is regarded as a problematic and common phenomenon in academic settings (Ziegler and Opdenakker, 2018). It is revealed that academic procrastination is a severe threat to academic success for college students (Jin et al., 2019).

Cluster 4 (Yellow): The largest node is performance, which concentrates on the ability and outcomes of study, such as students, learning disabilities, acquisition, intelligence ability, and higher education.

As shown in Table 8, the most popular topic in the area of academic procrastination is academic procrastination with the highest frequency keywords (142), followed by procrastination (104), self-regulation (31), academic performance (25), and college students (23). In Figure 6B, the density visualization map shows the hot spots of the research. Combined with the keywords findings, the hot topics of academic procrastination revolve around procrastination, academic procrastination, self-regulation, academic performance, and motivation. The top five keywords were analyzed, respectively, using 356, 273, 78, 68, and 110 papers by VOSviewer co-occurrence analysis. In the figure of overlay visualization, the size of the circle indicates the keyword weight, and the color refers to the average citation score of the keyword.

**Table 8:** Top 30 keywords of academic procrastination research based on the frequency.

**Rank**	**Frequency**	**Keyword**	**Rank**	**Frequency**	**Keyword**
1	142	Academic procrastination	16	13	Self-Efficacy
2	104	Procrastination	17	12	Academic self-efficacy
3	31	Self-regulation	18	12	Adolescence
4	25	Academic performance	19	12	Self-Control
5	23	College students	20	11	Students
6	19	Academic achievement	21	10	Perfectionism
7	19	Delay discounting	22	10	Self-esteem
8	19	Education	23	10	Sleep
9	17	Motivation	24	9	Children
10	16	Adolescents	25	9	Developmental delay
11	16	Time management	26	8	Active procrastination
12	16	University students	27	8	Health
13	14	Delay of gratification	28	8	Impulsivity
14	14	Higher education	29	8	Learning strategies
15	13	Gender	30	7	Delay

#### Procrastination

**Figure 7:**
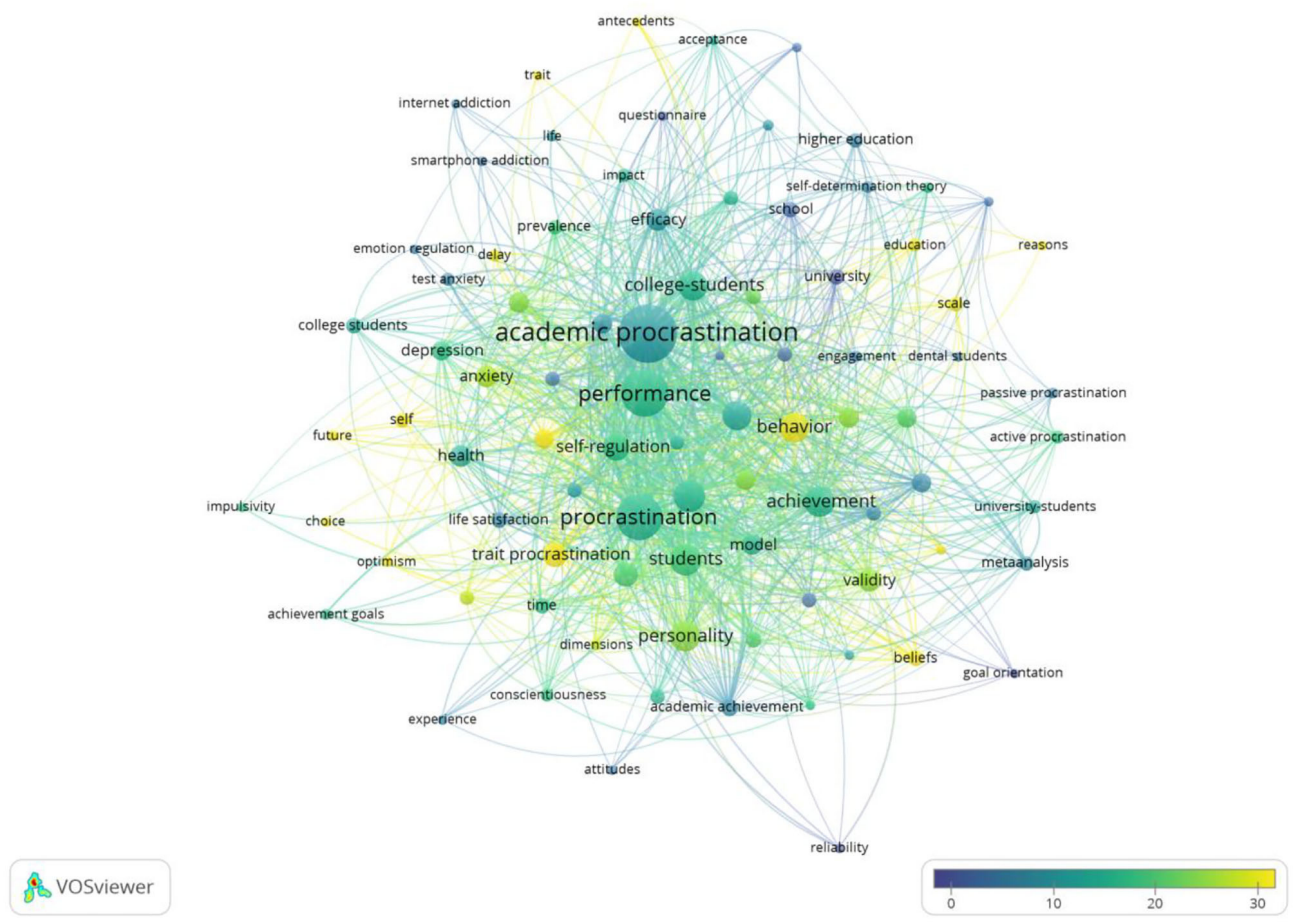
Overlay visualization based on procrastination link-weights and citation scores.

Procrastination means to “voluntarily delay an intended course of action despite expecting to be worse off for the delay” (Steel, 2007; Steel and Ferrari, 2013), which is very common among students throughout the educational process (Uzun Ozer et al., 2014). Studies show that as high as 95% of American students suffer from procrastination (Steel, 2007), and university students who have the habit of procrastination are more than 50% (Ferrari et al., 2005). Figure 7 shows that the keywords with more than 10 scores were behavior, self-esteem, delay, trait procrastination, academic performance, personality, perfectionism, and self-regulation. Analyzing the impact of academic procrastination on other factors is equally significant, especially its relationship with self-regulation and academic performance. Much research shows that students' propensity to procrastinate is an important factor affecting students' performance, so the existence of this behavioral tendency is that students with lower propensity to procrastinate usually get better grades than students with higher propensity to procrastinate (Hooshyar et al., 2020). A meta-analysis study shows that procrastination has a negative effect on academic performance (Kim and Seo, 2015). Recently, a study examined the effects of self-regulation on procrastination among students (de la Fuente et al., 2021), and a lot of research demonstrates that the relationship between procrastination and self-regulation are negative (Valenzuela et al., 2020).

#### Academic Procrastination

**Figure 8:**
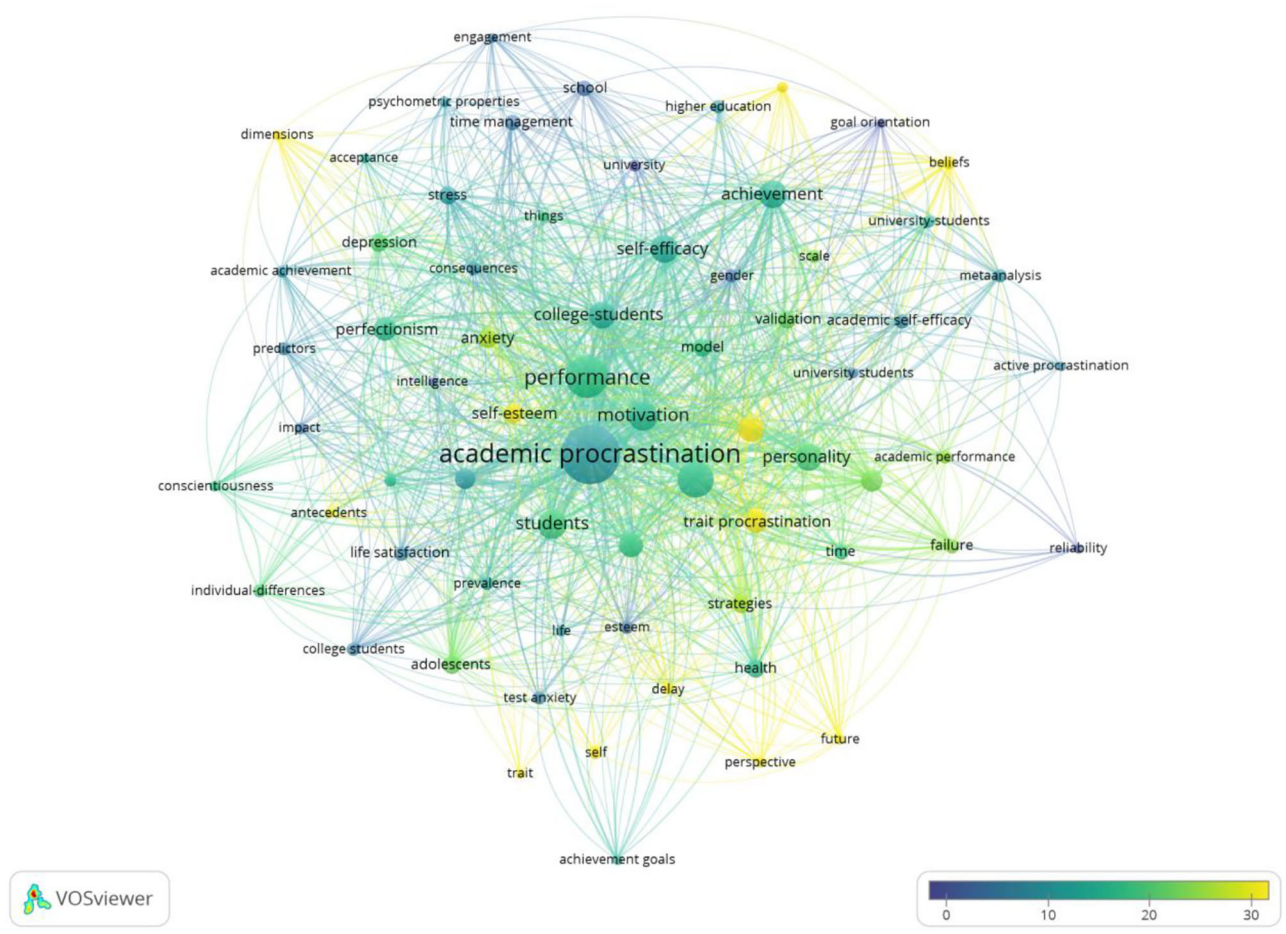
Overlay visualization based on academic procrastination link-weights and citation scores.

Based on an initial selection of 1,240 papers, 273 papers, accounting for 22%, are in connection with academic procrastination; this means that procrastination appears as a problematic phenomenon, especially in academic settings. When academic procrastination appears, academic goals are always put off, leading to psychological distress (Ferrari et al., 1995; Steel, 2007; Ljubin-Golub et al., 2019) and lower performance (Senécal et al., 1995; Schraw et al., 2007; Ljubin-Golub et al., 2019). As can be seen from Figure 8, the keywords appearing more than 10 times were self-regulation, failure, academic performance, delay, self-esteem, behavior, strategies, and perspective. Researchers investigated the interventions to overcome academic procrastination. Ziegler and Opdenakker study the effects of academic procrastination on effort regulation, self-efficacy, and metacognitive self-regulation (Ziegler and Opdenakker, 2018). A study inquired about the correlations between the ecological model of resiliency and adolescents' academic procrastination (Chen and Han, 2017). Toker and Avci revealed that skills training based on cognitive-behavioral theory reduced academic procrastination and had an impact on students for a long time (Toker and Avci, 2015).

#### Self-Regulation

**Figure 9:**
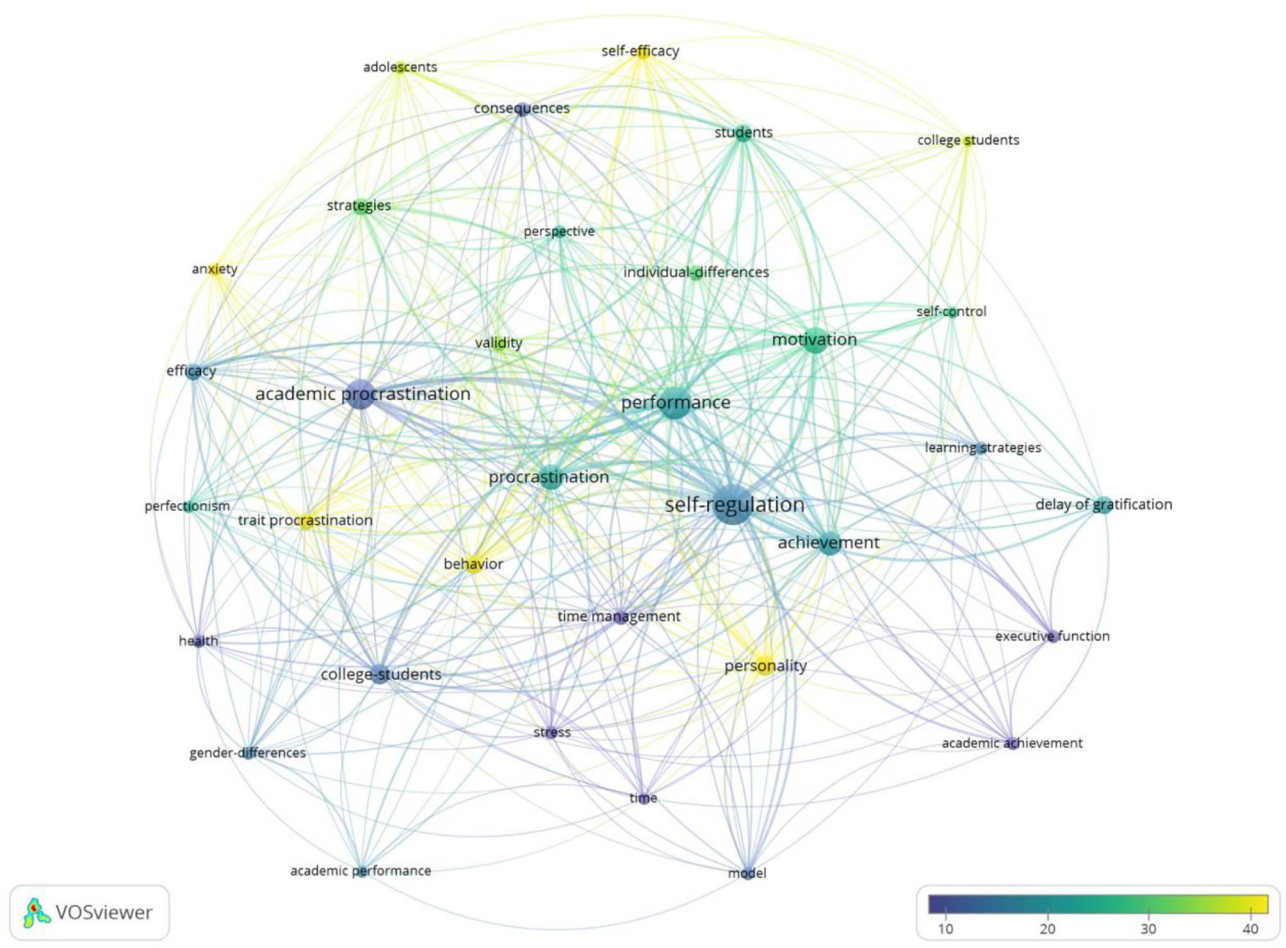
Overlay visualization based on self-regulation link-weights and citation scores.

Self-regulation is considered to be the meaning; construction of meta-behavioral order; and a skill for managing motivational, affective, and cognitive abilities (de la Fuente et al., 2017; Garzon-Umerenkova et al., 2018). Zimmerman's model shows that self-regulation consists of three cycle phases: forethought, performance, and self-reflection (Zimmerman, 2002; Loeffler et al., 2019). In Figure 9, the academic connections and emerging keywords are shown vividly. In the overlapping visual map, the keywords that appeared more than 10 times are self-efficacy, self-control, strategies, procrastination, performance, and motivation.

Self-regulation consists of subregulations such as self-efficacy, motivation, goals, cognitive, metacognitive, and resource management (Schmitz and Wiese, 2006). Self-efficacy in self-regulation has the most significant predictive effect on students' academic procrastination tendency, and it can prevent or reduce students' academic procrastination (Klassen et al., 2008; Zhang et al., 2018). Research verifies the effects of self-regulation on procrastination and confidence in academic behavior (de la Fuente et al., 2021) and reveals that lacking self-regulatory skills has a bad effect on college students' procrastination (Balkis and Duru, 2016).

#### Academic Performance

**Figure 10:**
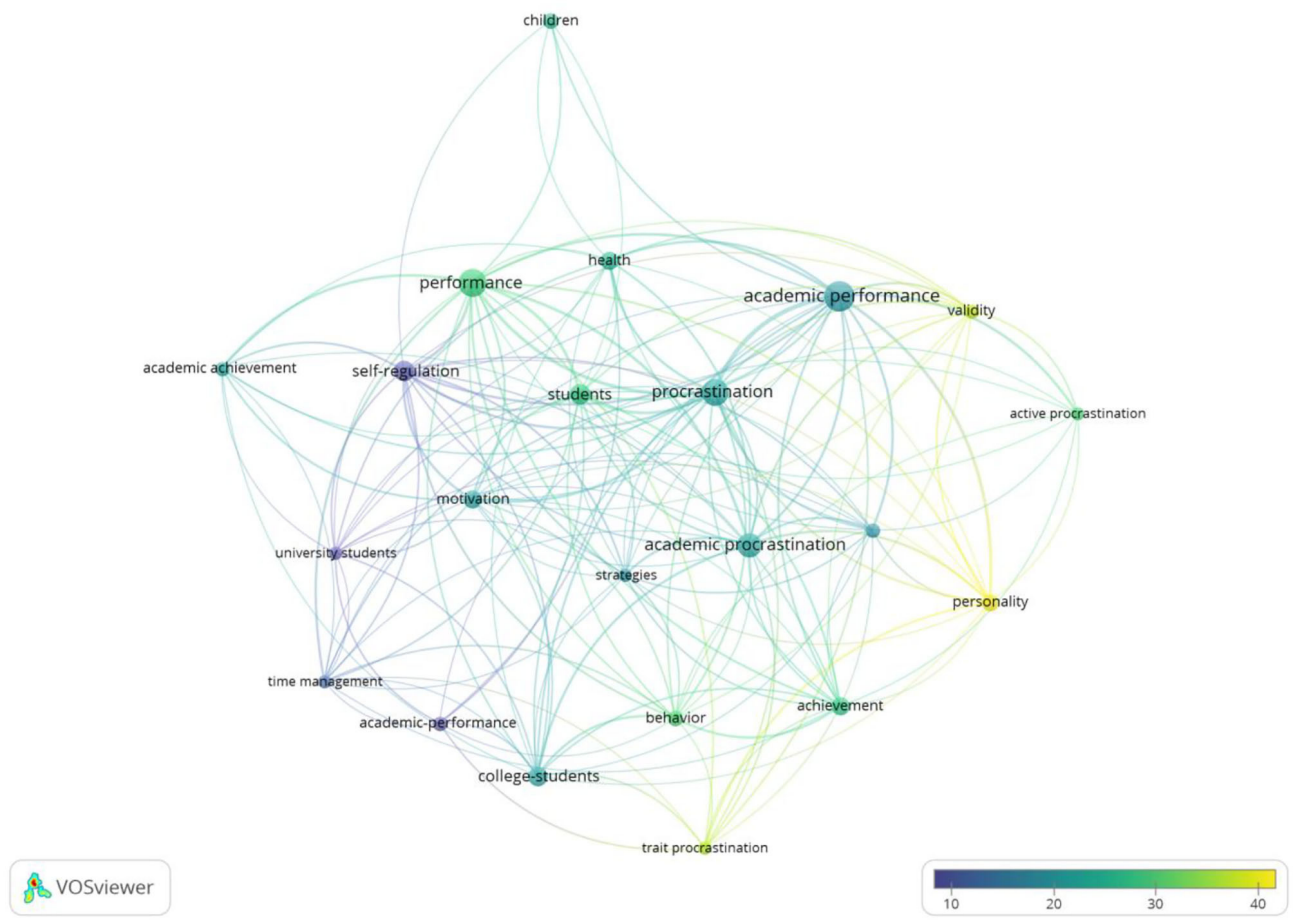
Overlay visualization based on academic performance link-weights and citation scores.

As shown in Figure 10, there exist significant correlations between academic performance and procrastination (including academic procrastination and active procrastination). In the overlapping visual maps, the keywords that appeared more than 10 times are academic procrastination, active procrastination, trait procrastination, personality, validity, self-regulation, motivation, and students. Prior research found that academic procrastination was a very important factor that decreased students' academic performance. A study tested whether the metacognitive model of procrastination could explain academic performance or not and showed that academic performance was impaired by procrastination (Fernie et al., 2018). Based on academic performance indicators, such as GPAs, test/assignment scores, quiz scores, and course grades, an analysis found that procrastination has a negative effect on academic performance (Kim and Seo, 2015; Sumaya and Darling, 2018). A study revealed that extraversion and neuroticism have a relationship with active procrastination. Furthermore, active procrastination can predict GPA better than passive procrastination (Kim et al., 2017). Research that examined irregular sleep times in relation to circadian rhythms and academic performance suggests that, for college students, light exposure patterns and irregular sleep have a relationship with decreased academic performance and delayed circadian rhythms (Phillips et al., 2017).

#### Motivation

**Figure 11:**
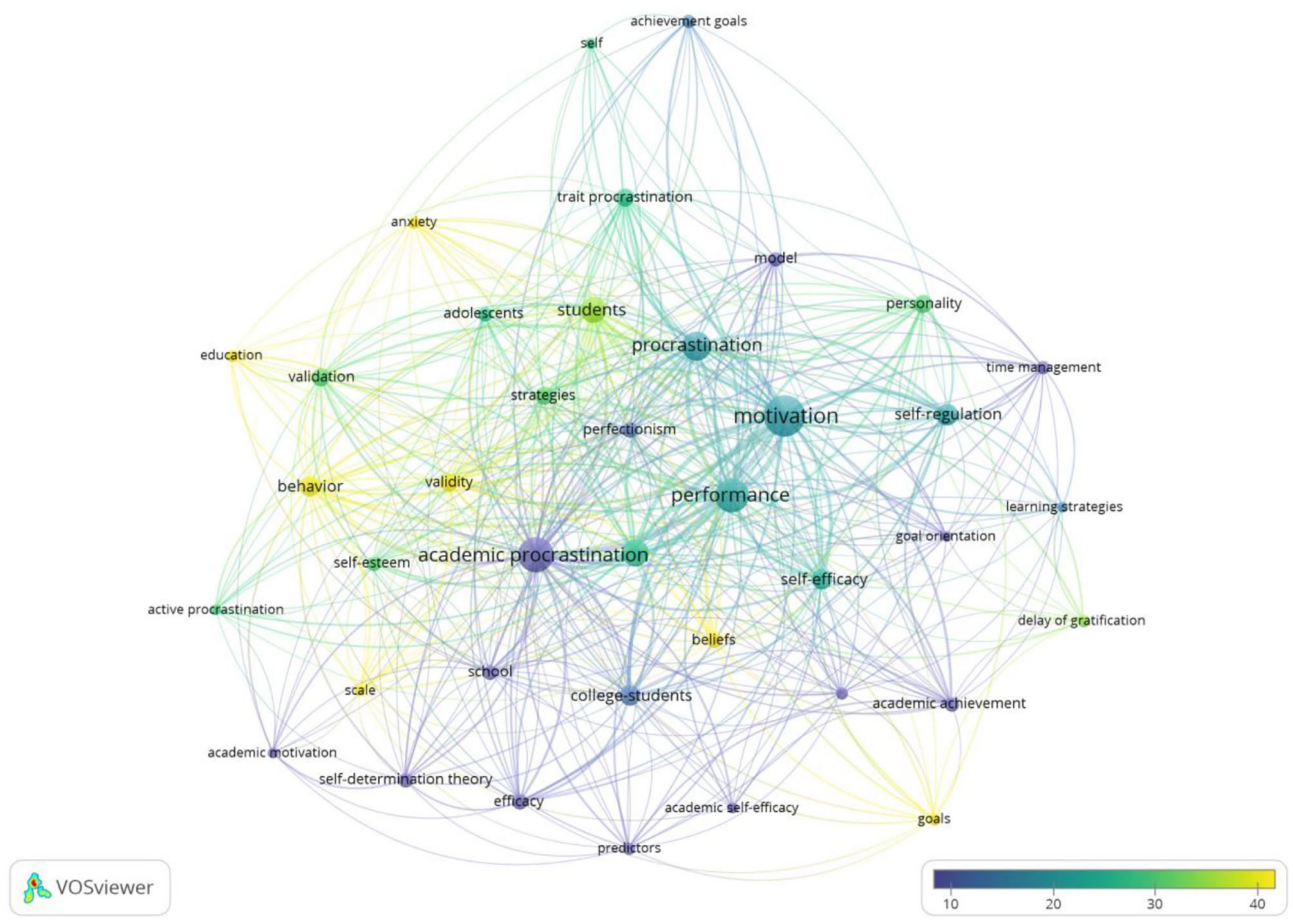
Overlay visualization based on motivation link-weights and citation scores.

Motivation can be considered to be a process to show great vigor and willpower so as to complete an assignment or task (Rakes and Dunn, 2010; Cavusoglu and Karatas, 2017). As can be seen from Figure 11, the keywords appearing more than 10 times are goals, anxiety, validity, self-esteem, performance, self-regulation, academic procrastination, time management, and academic self-efficacy. Previous findings show that motivation has a close relationship with other factors such as academic procrastination, academic achievement, and grades. Research using self-determination theory shows that procrastination has a negative effect on intrinsic (Rakes and Dunn, 2010; Wu and Fan, 2017) and extrinsic motivation (Reasinger and Brownlow, 2000; Wu and Fan, 2017). Similar findings reveal that motivation is related to academic procrastination significantly and negatively, and both of them were significant variables in predicting academic achievement (Akpur, 2017). The study conducting mediation analysis found that the stronger the self-determined motivation, the less procrastination and the more likely to achieve a higher GPA (Burnam et al., 2014).

## Conclusion

The present bibliometric paper offers an overall and visual review on the topic of academic procrastination. This study analyzes 1,240 valid articles about academic procrastination in the WoS core collection from the year 1938 to 2021. Since 1993, the publication of articles in academic procrastination has been rapidly increasing. Research in the area of academic procrastination is extensive and involves issues, such as a wide range of subjects, such as educational research, psychology multidiscipline, psychology educational, clinical neurology, and rehabilitation.

In terms of the analysis of cooperation between countries and institutions, the United States took a prominent lead among all countries, and Canada, Turkey, England, and China have all accomplished a fruitful job and achieved great contributions. Among the top 10 productive institutions, 8 out of 10 came from the United States. The truth reflects that the United States pays more attention to this field than any other countries do. The most productive institutions in this area were the University of Washington and University of California, Los Angeles. Additionally, international institutions and countries could enhance communication and cooperation with each other.

By analyzing the authors, it is clear that most authors usually like working with a few collaborators, leading to main groups of authors, such as Murat Balkis and June J. Pilcher. The most frequently cited author was Esther D. Rothblum, whose research analyzed how often college students procrastinate on academic assignments and reasons for their procrastinating behaviors.

Based on the co-citation journals network, *Personality and Individual Differences* was the prolific and influential journal referring to the number of citations and articles it received. In the past few years, *Personality and Individual Differences* has also attracted wide attention, which has given rise to the publishing process of academic procrastination. A lot of journals concentrate on psychology and multidisciplinary subjects and attract more and more researchers and scholars from all over the world.

The VOSviewer tool identified the hot spots of academic procrastination, which were mainly distributed as follows: (a) procrastination, (b) academic procrastination, (c) self-regulation, (d) academic performance, and (e) motivation.

## Recommendation

Several limitations exist in this bibliometric paper. First, the data is limited to articles only collected from the WoS database. Therefore, there is no other data from international databases, such as EBSCO, IEEE Xplore, Scopus, and Science Direct. Second, the keywords query of this bibliometric research was limited to a title search, so only 1,240 articles were analyzed. Future research could broaden the search range to titles, abstracts, and keywords to cover more related articles. Third, it should be noted that both the Bibliometix tool and VOSviewer software have their own limitations although many researchers have used the techniques in bibliometric paper, so it is better if other software is combined with them to analyze the data. Finally, the results of this study show that, in the area of academic procrastination, most authors like working with a small number of collaborators, which lead to few links between the major authors. Future researchers could work with more collaborators in doing their research. In conclusion, the analysis results are obviously stable and reliable and are almost not affected by subjective experience.

## Author Contributions

XT: analyzed the data and drafted this paper. HH: checked and modified the manuscript. HA: given the idea of the topic selection and the work design. NE: provided the detailed usage of software for analyzing. All authors contributed to the article and approved the submitted version.

## Conflict of Interest

The authors declare that the research was conducted in the absence of any commercial or financial relationships that could be construed as a potential conflict of interest.

## Publisher's Note

All claims expressed in this article are solely those of the authors and do not necessarily represent those of their affiliated organizations, or those of the publisher, the editors and the reviewers. Any product that may be evaluated in this article, or claim that may be made by its manufacturer, is not guaranteed or endorsed by the publisher.
